# *In vitro* study of decellularized rat tissues for nerve regeneration

**DOI:** 10.3389/fneur.2022.986377

**Published:** 2022-09-15

**Authors:** Kai Ye, Andong He, Miaoben Wu, Xiaodong Qiu, Zhiwu Chen, Jun Yin, Qinghua Song, Yi Huang, Kailei Xu, Yuye Huang, Peng Wei

**Affiliations:** ^1^School of Medicine, Ningbo University, Ningbo, China; ^2^Department of Respiratory and Critical Medicine, Ningbo First Hospital, Ningbo, China; ^3^Department of Surgery, Beilun Binhai New City Hospital, Ningbo, China; ^4^Department of Plastic and Reconstructive Surgery, Ningbo First Hospital, Ningbo, China; ^5^The State Key Laboratory of Fluid Power and Mechatronic Systems, School of Mechanical Engineering, Zhejiang University, Hangzhou, China; ^6^Key Laboratory of 3D Printing Process and Equipment of Zhejiang Province, School of Mechanical Engineering, Zhejiang University, Hangzhou, China; ^7^Medical Research Center, Ningbo First Hospital, Ningbo, China; ^8^Central Laboratory, Center for Medical and Engineering Innovation, Ningbo First Hospital, Ningbo, China; ^9^Key Laboratory of Precision Medicine for Atherosclerotic Diseases of Zhejiang Province, Ningbo, China

**Keywords:** peripheral nerve injury, decellularized tissue, bio-scaffolds, PC-12 cells, ECM

## Abstract

Peripheral nerve injuries cause an absence or destruction of nerves. Decellularized nerves, acting as a replacement for autografts, have been investigated in the promotion of nerve repair and regeneration, always being incorporated with stem cells or growth factors. However, such a strategy is limited by size availability. The potential application in heterotopic transplantation of other decellularized tissues needs to be further explored. In this study, rat decellularized kidney (dK) was selected to be compared with decellularized peripheral nerve (dN), since dK has aboundant ECM components and growth factors. The PC-12 cells were cultured on dK and dN scaffolds, as shown in the similar behaviors of cell metabolism and viability, but have a more regular arrangement on dN compared to dK, indicating that the natural structure plays an important role in guiding cell extension. However, we found significant upregulation of axon–growth–associated genes and proteins of PC-12 cells in the dK group compared to the dN group by qRT-PCR, immunofluorescence, and western blotting. Furthermore, various neurotrophic factors and growth factors of acellular kidney and nerve were evaluated by ELISA assay. The lower expression of neurotrophic factors but higher expression of growth factors such as VEGF and HGF from dK suggests that axon growth and extension for PC-12 cells may be partially mediated by VEGF and HGF expression from decellularized kidney, which further points to a potential application in nerve repair and regeneration.

## Introduction

Millions of people worldwide are injured in traffic, sports, and military accidents every year, accidents which commonly lead to peripheral nerve injuries (PNIs). The absence or destruction of nerves is one of the greatest challenges that novel therapeutic options face in neural functional restoration through nerve regeneration, a complicated biological process that begins almost immediately following nerve injury. Normally, direct suture and autologous nerve transplantation are the most commonly used clinical treatments, in particular autologous nerve transplantation, which serves as the gold standard (1, 2), providing the most complete regenerative environment for regenerating axons. However, autografts still suffer limitations including nerve unavailability, size mismatch, and local tissue adhesion (3–5).

Tissue engineering (TE) has paved the way for a new approach to PNI treatment. Artificial nerve conduits, for example, can be used to link the two ends of wounded nerves to support and encourage nerve regeneration (6–8). This is always characteristic of low antigenicity, supports vascularization, is porous for oxygen diffusion, and avoids long-term compression and tissue adhesion. Nerve-guiding conduits made of various materials have been previously investigated, and their ability to facilitate peripheral nerve regeneration has been demonstrated in animals (9, 10). Not only natural biomaterials, such as collagen (11), silk (12), and gelatin (13), but also synthetic neural conduits such as polylactic acid and poly (lactic-co-glycolic acid) (14, 15), play an important role in nerve regeneration with additional cells or growth factors. Today, decellularized tissue has been investigated as another alternative biomaterial for the treatment of peripheral nerve injury (16). Decellularized tissue, created by removing cellular components from native tissue, has been linked to reduced immunological rejection and is also a suitable microenvironment for cellular growth. Moreover, decellularized tissue involves many various extracellular matrix (ECM) components. It has normally been thought to be an inert structure that provides a platform for cell adhesion, but its effect on tissue repair coordinating growth factors, cytokines, and chemokines has been proven. Thus, the use of decellularized tissue for peripheral nerve injury is a potential treatment for nerve regeneration.

Currently, of the different decellularized ECM (dECM) materials, decellularized nerve tissue is the one most commonly used in nerve regeneration due to its homolog. The FDA has approved the use of acellular nerve tissue in clinical trials as a commercial product called Avance Nerve Graft, which has been implanted in patients all over the world with positive reparative results and no infection (17). However, for longer peripheral nerve deficits (>20 mm), the regeneration effectiveness of decellularized nerve tissue is still suffer the limitation of size availability (18). Decellularized kidney has been widely investigated for renal regeneration since it allows the injured area to re-cellularize with autologous stem cells or differentiated cells (19). Theoretically, decellularized tissue has relatively low heterogeneity because the cells are all removed. This provides for its potential application in heterotopic sites, such as when the decellularized small intestinal submucosa has been widely used in bone tissue engineering (20). However, few studies have used decellularized kidney in nerve regeneration—a potential application to be investigated.

In this study, we describe rat pheochromocytoma (PC-12) cell behaviors after culturing on a xenogeneic-free matrix derived from rat kidney and peripheral nerve. The efficiency of decellularization was examined by H&E staining, DNA quantification, and immune blotting and further sections were cut for cell culturing. In order to examine their biocompatibility, biodegradability, and nutritional factors existing, we investigated the proliferation rates of PC-12 cells with Cell Counting Kit-8 (CCK-8) and further quantified the live/dead cells. The mRNA level of functional genes was analyzed by qPCR. In addition, the morphology of PC-12 cells was determined by immunofluorescent staining. Further, various growth factors were evaluated by ELISA and immune blotting, respectively. These data indicate that diverse acellular, tissue-derived matrices provide a different microenvironment to enable the proliferation, expansion, and gene expression of neurocytes *in vitro*, which provides biomaterial selection in nerve repair and regeneration.

## Materials and methods

### Materials

High differentiated PC-12 cell was purchased from the Cell Bank of the Chinese Academy of Science (Shanghai, China). Roswell Park Memorial Institute (RPMI) 1640 Medium and 1% penicillin/streptomycin (P/S) were obtained from Gibco (USA). The Cell Counting Kit-8 (CCK-8) was purchased from Beyotime (C0039, China). The H&E Staining Kit, Live/Dead Viability Assay Kit, 4′, 6-diamidino-2-phenylindole (DAPI), antibodies Neurofilament heavy polypeptide (ab207176), GAP-43 (ab16053), donkey anti-mouse IgG H&L (ab175700), goat anti-rabbit IgG (ab150077), and the ELISA Kit of GDNF (ab213901) and FGF1 (ab223587) were obtained from Abcam (UK).The ELISA Kit of HGF (EK3H01-96), VEGF (EK383/2-96), and BDNF (EK3127-96) were obtained from LIANKE BIO (Hangzhou). GAPDH (RM2002), Goat anti-Rabbit IgG (RM3002), and Goat anti-Mouse IgG (RM3001) were bought from Ray Antibody Biotech (Beijing). All the primers were synthesized by Sangon Biotech (China).

### Fabrication of the decellularized matrix

Male Sprague-Dawley (SD) rats initially weighing 200 g, certification number SYXK (Zhejiang) 2013-0191, were used. The rats were anesthetized with an intraperitoneal injection of 10% chloral hydrate of a lethal dose (Wabcan, ST1002, China). After skin preparation, the bilateral kidneys and sciatic nerves were obtained and were cut into tissue pieces of a suitable size. The specimens were washed with PBS, stirred in 2% Triton X-100 in deionized water at room temperature for 24 h, and the solution was changed each 12 h. Then, the specimens were stirred in 0.1% sodium dodecyl sulfate (SDS, L3771-500G, SIGMA, US). Finally, after washing with deionized water for 24 h, decellularized tissues were obtained and stored at −80°C.

To determine the extent of decellularized tissues, GAPDH was detected by western blot analysis, histological species were analyzed by H&E staining, and the residual DNA of the decellularized tissues was measured using the TIANamp Genomic DNA kit.

### Porosity measurement

The porosity of decellularized tissues was measured with a modified liquid displacement method using isopropanol (21, 22), since it can penetrate into the pore structure of decellularized tissues without inducing shrinkage or swelling. Briefly, the decellularized tissues were cut into similar shapes and the dimensions were measured with a digital micrometer (Mitutoyo, Japan) after freeze-drying. The decellularized tissues were soaked in isopropanol with a density (ρ) of 0.785 g/mL under vacuum for 30 min and continue soaking in a 50 ml tube overnight for better penetration. The samples were taken out from isopropanol and weighted immediately to avoid evaporation. The porosity was calculated using the following equation:


(1) $$\begin{array}{r} Porosity=\frac{\left(W2-W1\right)/\rho }{V}\times 100\% \end{array}$$

where W2 is the weight of decellularized tissue after immersion, W1 is the weight of decellularized tissue before immersion, V is the volume of hydrogel after immersion, and ρ is the density of isopropanol.

### SEM images and pore-size measurement

The decellularized tissues were imaged using a scanning electron microscope (Phenom Pro, Netherlands) at 2000×. The images were analyzed using *NIH ImageJ* to count the average pore size of the decellularized tissues.

### Preparation of cell slides and frozen-tissue sections

The coverslips (12 mm diameter) were soaked in poly-lysine solution (PLL, 10-fold dilution in sterile deionized water) at room temperature for 5 min and dried in an oven (BLUEPARD, DHG-9030, Shanghai) at 60°C for 1 h.

The decellularized kidney and nerve were flattened in a cryosection box with an area of about 0.2 cm^2^ and were then embedded in optimal cutting temperature compound (O.C.T. Compound, SAKURA, USA) and frozen at −20°C for 1 h. The decellularized tissues were cut into slices with a thickness of 50 μm and affixed to the pretreated cell slides. We peeled off the O.C.T. Compound after drying it at room temperature for 30 min with a fine forceps and then put the cell slides into a 24-well plate. We washed the slides with alcohol and PBS. After soaking in PBS for 12 h, the O.C.T. Compound was completely removed. Finally, the slides were sterilized with a UV sterilizer for 60 min.

### Cell culture

The PC-12 cells were cultured in RPMI-1640 supplemented with 10% fetal bovine serum (FBS, Excell Bio, FCS500, USA) and 1% P/S. The cells were cultured in a humidified incubator (Thermo Scientific, USA) at 37°C and 5% CO_2_. The cells used for experiments were in a logarithmic growth phase and P1 to P10 generation.

The cell slides attached to the decellularized tissue were placed in the bottom of the 24-well plate, and each well was seeded with 5000 PC-12 cells. In order to compare the accretion rate of the cells on the different decellularized tissues, the cells were observed using a microscope (Leica, Germany) at 1, 3, 5, and 7 days.

### Live-dead assay

A fluorescence live/dead assay was performed based on the manufacturer's protocol to compare the viability of the cells. First, samples were washed 3 times with a 1× assay buffer from a kit and then incubated with 500 μL of working solution (2 μM Calcein-AM and 4.5 μM PI) for 15 min in an incubator. The stained samples were then washed 3 times using a 1× assay buffer. Finally, we used an inverted phase-contrast fluorescence microscope (Leica, Germany) for imaging and used *ImageJ* to quantify the resulting images (*n* > 3) for dead-cell analysis.

### Immunofluorescent assay

The protein expression of the PC-12 cells was detected by immunofluorescence staining. The samples were fixed in 4% PFA for 2 h, washed with PBS, permeabilized with 0.5% TritonX-100 for 15 min, and closed with closure buffer (3% BSA) for 2 h at room temperature. The primary antibody of the PC-12 cells was NF-H (1:100) and GAP-43 (1:100). After incubation overnight at 4°C, the donkey anti-mouse IgG H&L (1:500, Alexa Fluor^®^ 568) and goat anti-rabbit IgG (1:500, Alexa Fluor^®^ 488) were incubated for 1 h at room temperature, respectively. Cell nuclei were stained with DAPI, and the samples were washed with PBS three times and then imaged by an inverted phase-contrast fluorescence microscope (Leica, Germany).

Phalloidin assay was carried out on days 3, 5, and 7 to analyze the cellular morphology of the PC-12 cells on decellularized tissues. First, the samples were fixed with 4% PFA for 10 min at room temperature and then rinsed out with PBS. Next, the cells were permeabilized with 0.5% TritonX-100 for 10 min at room temperature. Phalloidin solution (Solarbio, China) was applied for further incubation in the dark for 2 h followed by DAPI staining for nuclei for 5 min. After washing away the solution, the morphological structure of the cells was imaged with confocal microscopy (Nexcope, China).

### Cellular proliferation analysis

CCK-8 assays were performed on days 1, 3, 5, and 7 to compare the proliferation of PC-12 cells. “CCK-8 assays were performed according to the manufacturer's protocol. At each time-point, all groups of samples (*n* = 4) were transferred to a new 24-well plate, added with 350 μL of 10% CCK-8 solution diluted with fully supplemented RPMI-1640 medium, incubated in 37°C for 2 h. After incubation, 200 μL of the 10% CCK-8 solution was transferred into a 96-well plate and measured with absorbance at 450 nm using a microplate reader (BIO-RAD, USA).”

### Real-time PCR

PC-12 cells were seeded on the decidualized tissues in 24-well plates and cultured to 80% confluence. Then the cells were harvested, and RNA was extracted using TRIzol (Life, USA) for qRT-PCR analysis of GAPDH, Ki67, CAMK2A, NF-H, and GAP-43 mRNA levels. Further separation was carried out using chloroform and they were washed with isopropanol and 75% ethanol. Finally, the extracted RNA was dissolved in RNase-free water, and its concentration was measured with Nanodrop One (Thermo Scientific, USA). Complementary DNA (cDNA) was synthesized using the FastKing gDNA Dispelling RT SuperMix kit (TIANGEN, China). Relative gene-expression analysis was assessed using real-time PCR (Roche, Switzerland). Power SYBR Green PCR Master Mix (Life, USA) was mixed with 50 ng cDNA and specific primers (Table 1) in a total volume of 10 μL.

**Table 1:** Primer sequence.

**Primer name**	**Sequence (F) 5^′^-3^′^**	**Sequence (R) 5^′^-3^′^**
ACTIN	CCGCGAGTACAACCTTCTTG	CAGTTGGTGACAATGCCGTG
GAP43	CCGACAGGATGAGGGTAAAG	GCAGGAGAGACAGGGTTC
NF-H	AAGGAAACCGTCATTGTAGAGGAA	GGAGACGTAGTTGCTGCTTCTT
CAMK2A	GATGTGCGACCCTGGAATGA	ATGTAGGCGATGCAGGCTGAC

### Western blotting

Cells on decidualized kidneys and nerves were cultured to 80% confluence, collected, and lysed on ice for 20 min using RIPA lysis solution. Cell lysates were centrifuged for 15 min at 13,000 rpm. The protein concentration in the supernatant was determined using a BCA kit (Thermo Scientific, 23225, USA). About 15 μg of protein was separated with sodium dodecyl sulfate-polyacrylamide gel electrophoresis (SDS-PAGE) and transferred to polyvinyl difluoride (PVDF) membranes, which subsequently were blocked using 5% non-fat milk at room temperature for 1 h. Next, the primary antibodies NF-H (1:1000), GAP-43 (1:1000), and GAPDH (1:1000) were used for PC-12. After washing with 0.05% TBST three times, the membranes were incubated with secondary antibodies, Goat anti-Rabbit IgG (1:5000), and Goat anti-Mouse IgG (1:5000) for 2 h at room temperature. Finally, the membranes were incubated with chemiluminescent substrate (BIO-RAD, 1705061, USA) and transferred to the Clarity Western ECL substrate detection solution (BIO-RAD, USA) to visualize protein signals. The intensity of the protein bands was measured using *ImageJ*. GAPDH was used as an internal reference.

### ELISA

The levels of HGF, VEGF, BDNF, GDNF, and FGF1 were measured in decellularized tissues using an ELISA Kit based on the manufacturer's protocol. The decellularized tissues were crushed using a homogenizer (JXFSTPRP-64L, Jingxin, Shanghai). The tissue homogenates were centrifuged to obtain supernatants and analyzed using a microplate reader (Molecular Devices, SpectraMax iD3, USA).

### Statistical analysis

Unless otherwise stated, all the results were analyzed using *GraphPad Prism 9*. All the experiments were performed in triplicate. Data are expressed as mean ± standard deviation. Student's *t*-tests was used for all data. A *P* < 0.05 was considered a statistically significant difference.

## Results

### Decellularized efficiency testing

**Figure 1:**
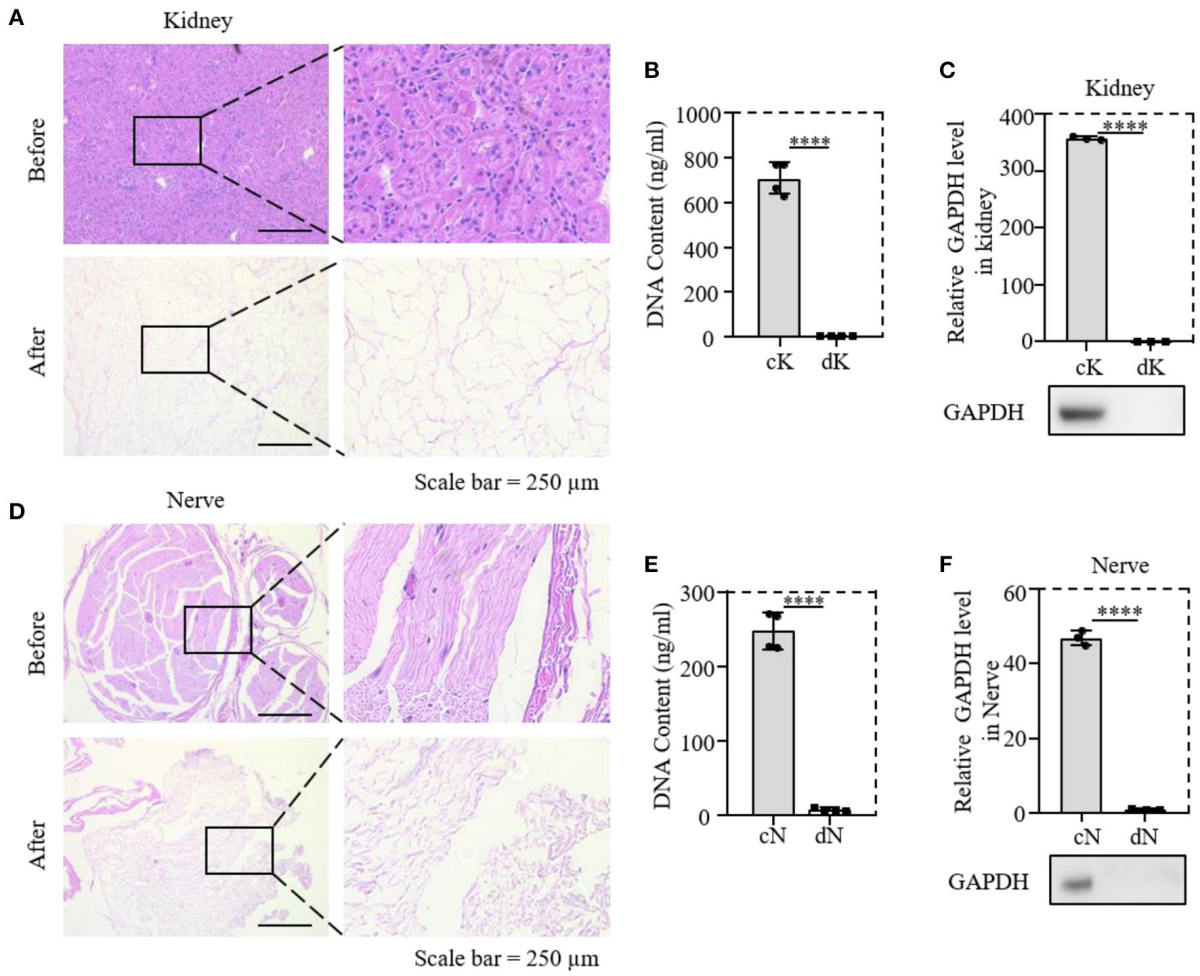
Validation of the decellularized efficiency of kidney and nerve tissue; **(A,D)** comparison of H&E staining before and after decellularization of rat tissues; **(B,E)** comparison of DNA content in tissues before and after decellularization; **(C,F)** relative protein expression of GAPDH; cK, control kidney; dK, decellularization kidney; cN, control nerve; dN, decellularization nerve. ****P ≤ 0.0001.

To prove the decellularization efficiency of our protocol, the rat kidney and nerve were evaluated by H&E staining to demonstrate cytoplasm and cell nucleus removal. Both the kidney and nerve tissues before decellularization were termed control kidney and control nerve (cK and cN), and decellularized tissues were termed dK and dN. It was obvious that nucleus and cytoplasm were almost removed with the natural scaffold being retained (Figures 1A,D). DNA quantification and measurement of the protein level of GAPDH were further performed for both decellularized tissues (Figures 1C,F). Quantitative analysis (Figures 1B,E) of decellularization efficiency shows that the DNA content decreased from 670 to 5 ng/mg in renal tissues. In neural tissues, the DNA content decreased from 165 to 3 ng/mg. The DNA-content determination results show that the DNA removal rate of neural tissues reached 96.62 ± 0.61% and was more than 99.17 ± 0.23% in renal tissues. GAPDH is an enzyme in the glycolysis process and acts as a housekeeping gene expressed at high levels in almost all tissues. The relative protein expression of GAPDH in renal tissues and neural tissues almost dropped to 0 (Figures 1C,F).

### Material characterization of dK and dN

**Figure 2:**
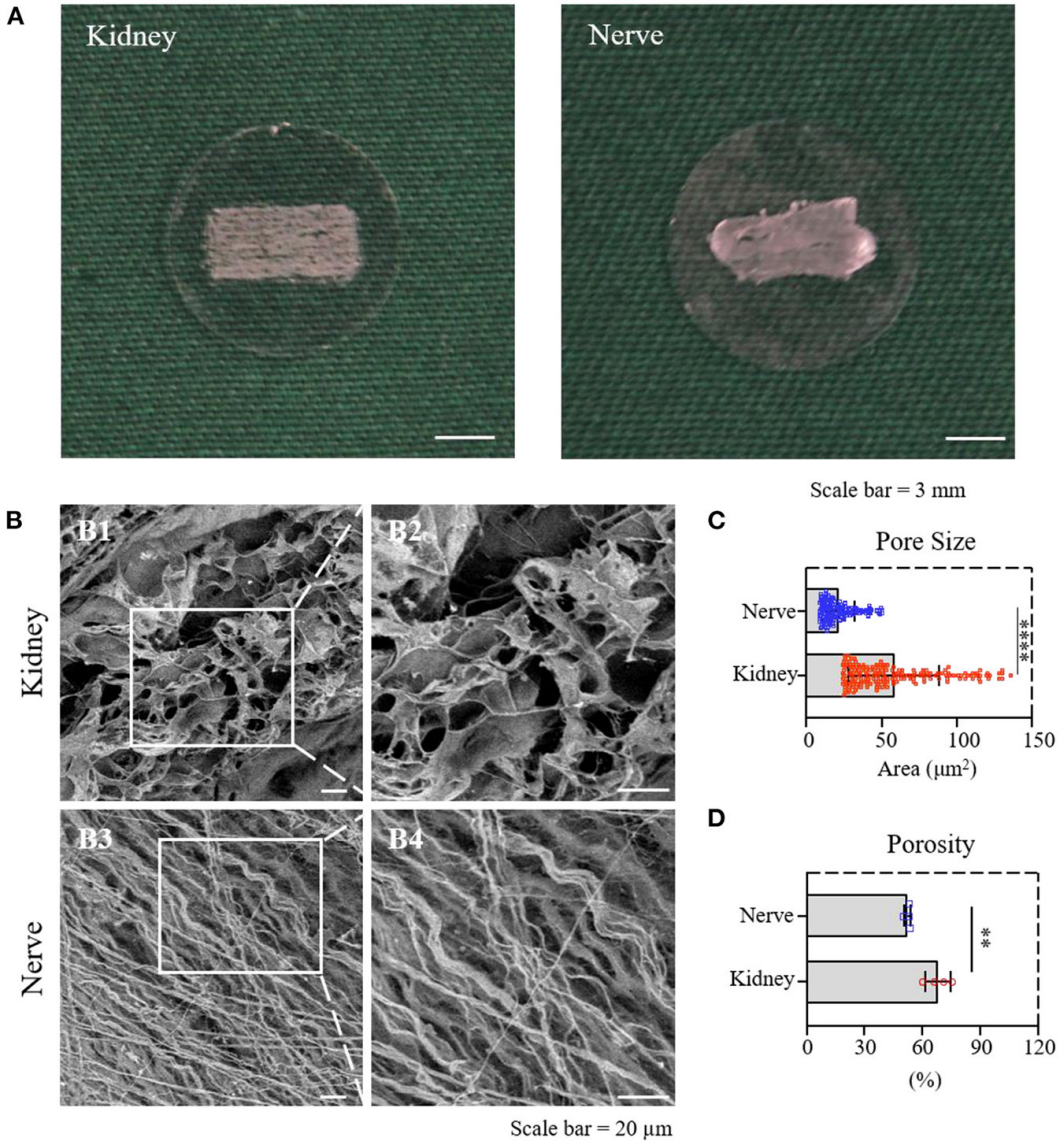
Material characterization of dK and dN; **(A)** The decellularized frozen-tissue species on the cell slides; **(B)** SEM images showing the three-dimensional microarchitectures of decellularized kidney and nerve; **(C)** statistical result of the pore area of decellularized kidney and nerve; **(D)** statistical result of the porosity of decellularized kidney and nerve. **P ≤ 0.01 and ****P ≤ 0.0001.

dK and dN were cut into slices with a 50 μm thickness with similar sizes and attached to PLL-coated 24–well-plate coverslips (Figure 2A) to create the cell culture in the following experiments. The surface structure of dK and dN was imaged with SEM, in which dK demonstrated an interconnected pore structure (Figure 2 B1,B2), while dN had a fiber-like structure (Figure 2 B3,B4). The pore sizes for dK and dN were futher analyzed using *NIH ImageJ*. dK had a significantly larger pore size compared with dN, which were 58.13 ± 30.10 and 21.10 ± 10.70 μm^2^, respectively (Figure 2C). The porosity was measured using the liquid substitution method. Similar in pore size, dK had significantly higher porosity than dN-−68.18 ± 6.50 and 52.50 ± 1.68%, respectively (Figure 2D), suggesting that dK could provide better cell migration and nutrition diffusion when applied *in vivo*.

**Figure 3:**
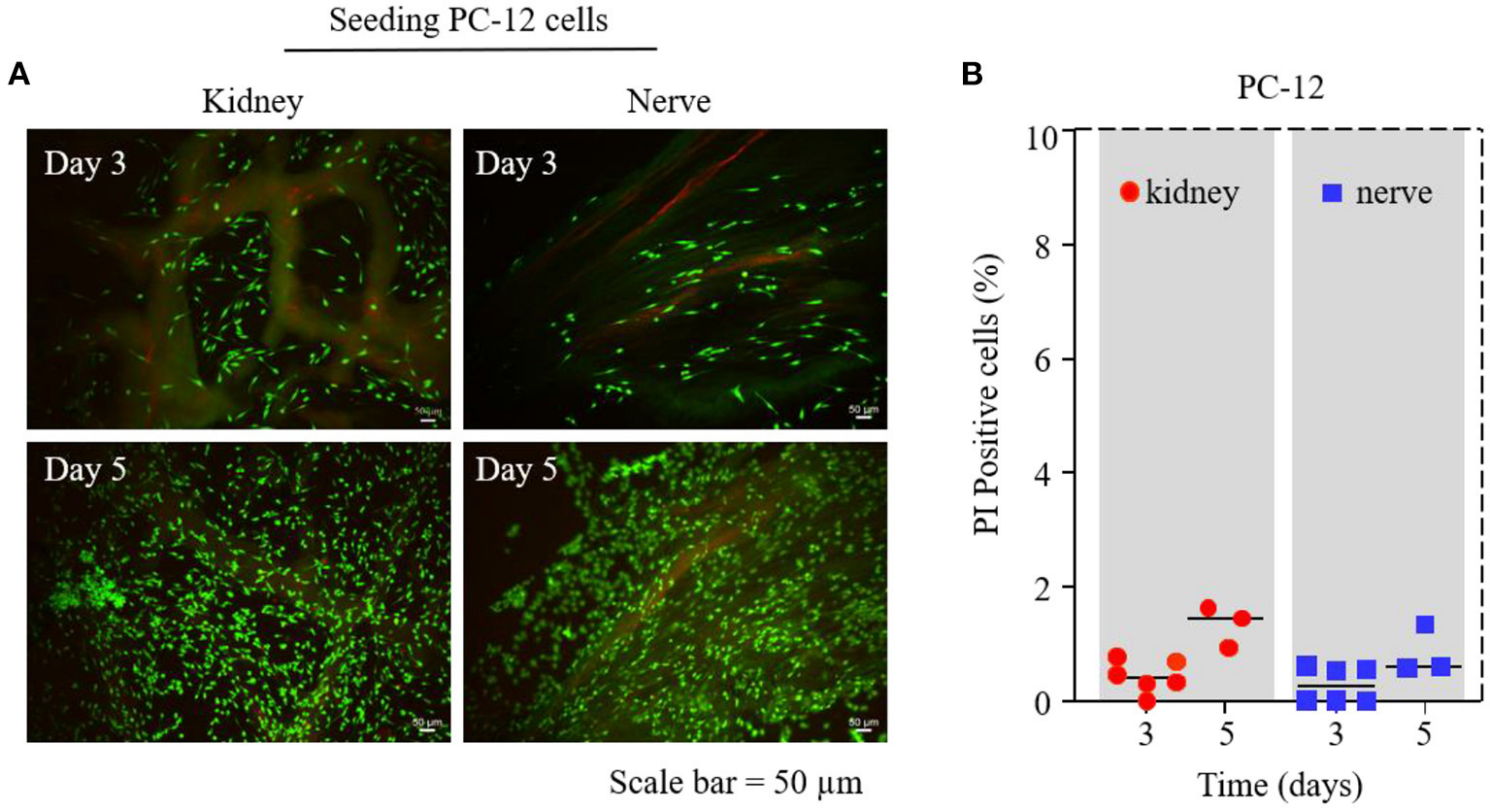
PC-12 cell survival test on dK and dN; **(A)** Live/Dead assay by Calcein-AM and PI staining on days 3 and 5—living cells are depicted in green and dead cells are in red; **(B)** statistical cell-viability quantification of PI-positive cells (dead cells).

**Figure 4:**
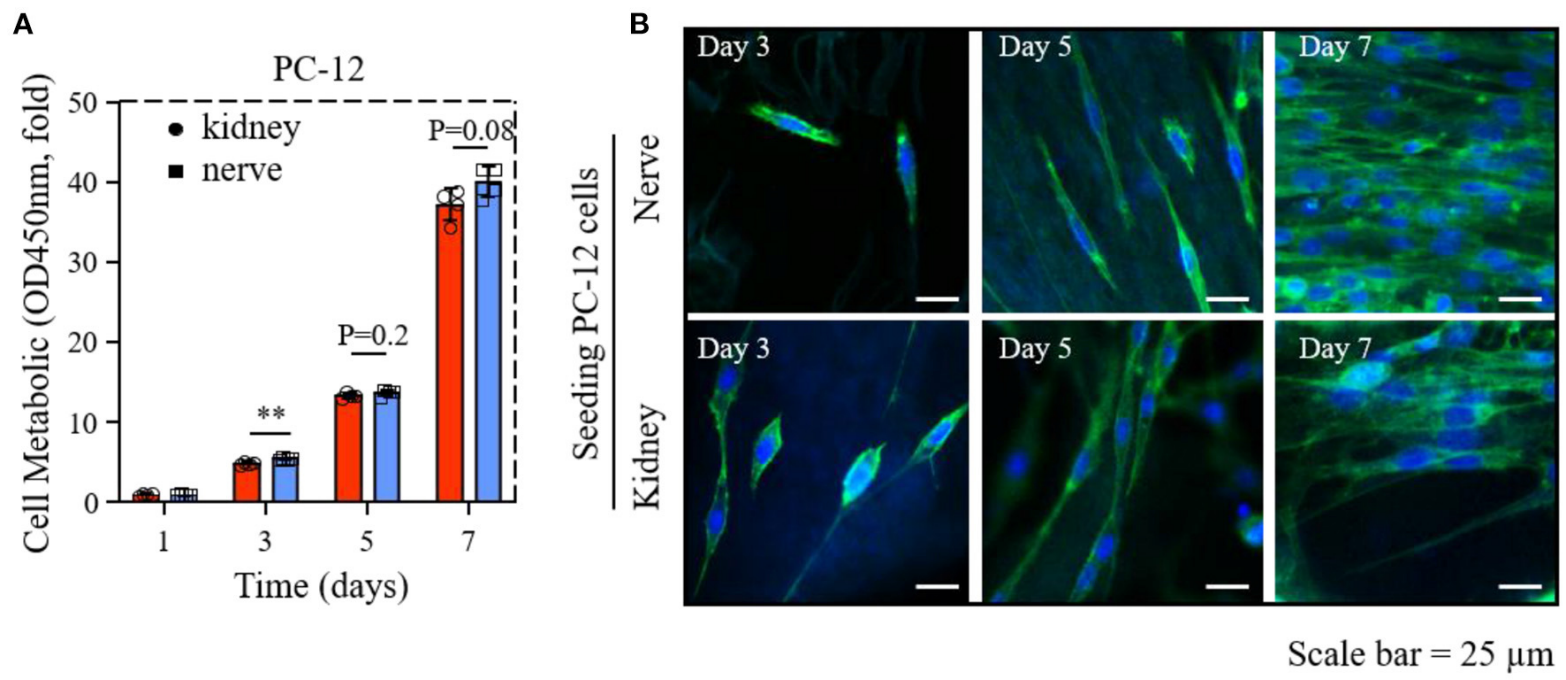
The proliferation and morphology of PC-12 cells cultured on dK and dN; **(A)** The cck-8 result of PC-12 cells in 1, 3, 5, and 7 days; **(B)** images of PC12 cells stained with phalloidin (green) and nucleus (blue) after 3, 5, and 7 days of culture. **P ≤ 0.01.

### The proliferation, viability, and morphology of PC-12 cells seeding on dECMs

We used a Live/Dead viability assay on days 3 and 5 to investigate the influence of different dECMs on PC-12 cell viability. Figure 3A shows that there were no significant differences for the viability of PC-12 cultured on dK and dN group. Dead cells were counted to further quantify cell viability, where the results shown in Figure 3B demonstrated that the number of dead PC-12 cells on dK and dN were both <2%, suggesting that both dK and dN have excellent biocompatibility.

The results of the CCK-8 assay of PC-12 cells on dECMs shows that dK and dN successfully supported cellular growth. The cellular proliferation rates of PC-12 cells seeded on dK were 4.7–, 13.2–, and 36.5–fold changes for days 3, 5, and 7, respectively, normalized to day 1 (Figure 4A). The PC-12 cells grew slowly from day 3 to day 5 but grew significantly faster from day 5 to day 7. Meanwhile, the cellular proliferation rates of PC-12 cells cultured on dN demonstrate a similar tendency with an increasing change of 5.2–, 13.6–, and 38.5–fold for days 3, 5, and 7, respectively, compared to day 1 (Figure 4A). Overall, PC-12 cells demonstrated a similar cell proliferation rate on both dECMs at all time-points, even though PC-12 cells on dN at day 3 showed a slightly higher proliferation rate than on dK (*P* < 0.1).

Phalloidin/DAPI staining was used to investigate the influence of dECMs on PC-12 cells morphology. The morphology of PC-12 cells on both two dECMs were randomized due to fewer cells on day 3 and became significant ordered arrangement from day 5 to day 7, which may result from the renal and neural morphology (Figure 4B). Obviously, both dECM scaffolds show support for extensive morphology of PC-12 cells at all time points (Figure 4B).

Based on the cell proliferation and viability results, both rat kidney and peripheral nerve ECM provided a better microenvironment for PC-12 cells growth.

### Axon-extension-related gene expression

**Figure 5:**
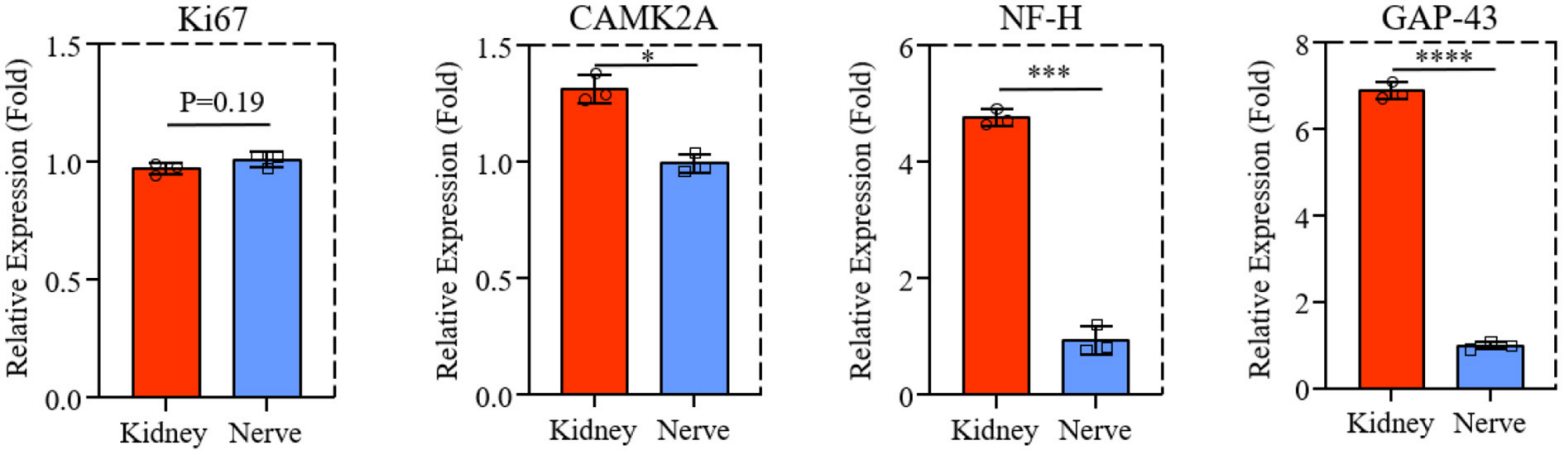
The qPCR result of Ki67, CAMK2A, NF-H, and GAP-43 expression of PC-12 cells cultured on dK and dN. *P ≤ 0.05, ***P ≤ 0.001, and ****P ≤ 0.0001.

The mRNA level of Ki67, CAMK2A, NF-H, and GAP-43 gene expression from PC-12 cells on dK and dN were analyzed by qPCR to investigate the advantages of dECM scaffolds. As shown in Figure 5, the proliferation-related gene Ki67 for PC-12 cells did not change much between dK and dN (*P* = 0.19). However, the CAMK2A, NF-H, and GAP-43 gene expression for PC-12 cells significantly increased in dK, which represents axon growth and extension when compared with cells cultured on dN.

### dECMs promote axon extension-related protein expression

**Figure 6:**
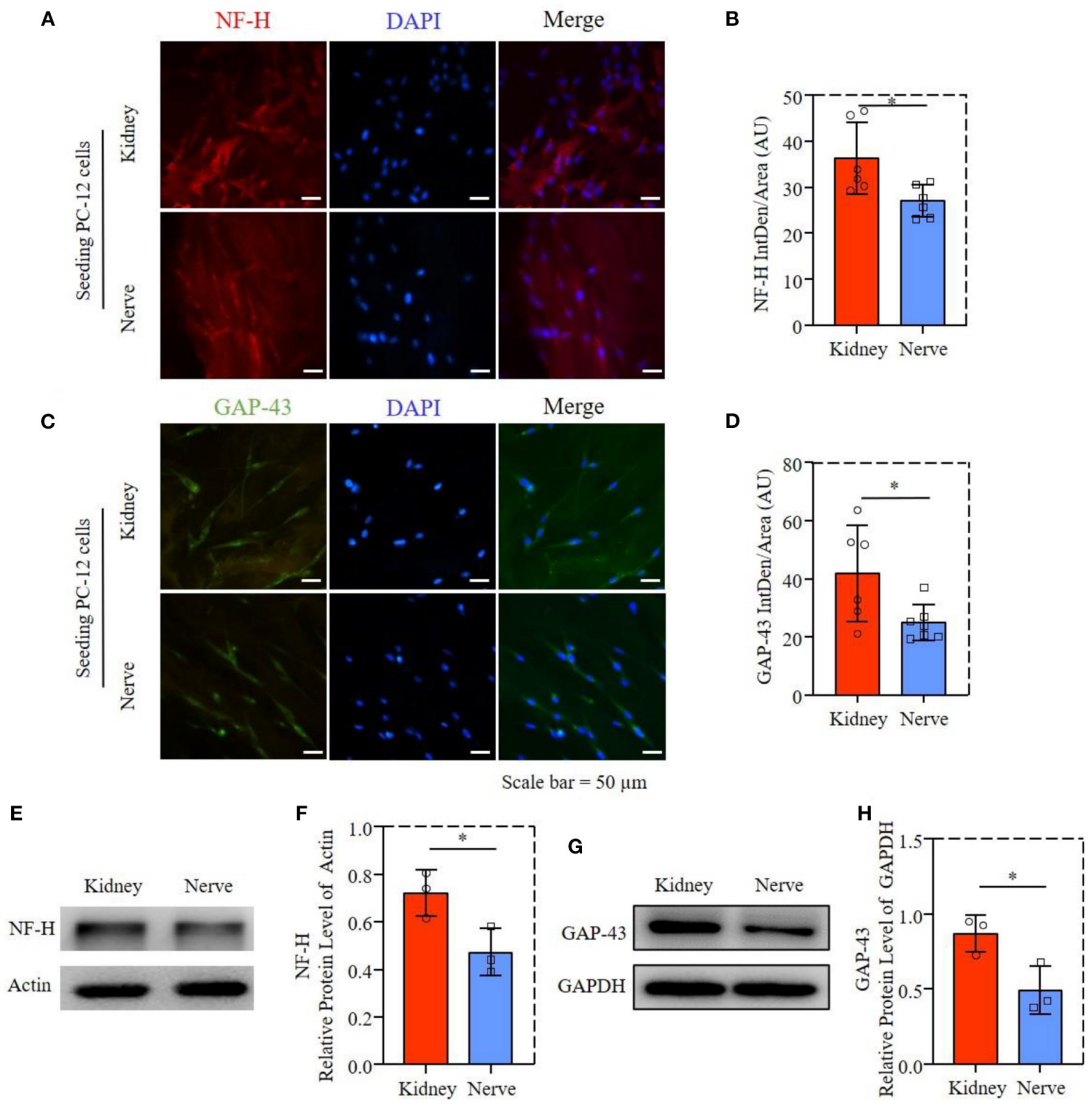
Axon extension-related proteins expression of PC-12 cells; **(A)** Fluorescence microscopy images of PC-12 cells stained with NF-H (red) and cell nuclei (blue). **(B)** Statistical fluorescent intensity of NF-H. **(C)** Fluorescence microscopy images of PC-12 cells stained with GAP-43 (green) and cell nuclei (blue). **(D)** Statistical fluorescent area of GAP-43. **(E)** The NF-H in PC12 cells was determined by western blotting. **(F)** Relative protein expression of NF-H. **(G)** The GAP-43 in PC12 cell was determined by western blotting. **(H)** Relative protein expression of GAP-43. *P ≤ 0.05.

The axon proliferation-related proteins of NF-H and GAP-43 were performed by immunofluorescence staining, as shown in Figures 6A,C. The PC-12 cells had similar axonal extension on both dECMs, while NF-H and GAP-43 expression of PC-12 cells were slightly higher in dK than in dN, with the quantification results of fluorescence intensity in Figures 6B,D. This observation was further supported by western blotting (Figures 6E,G), as well as quantification results shown in Figures 6F,H, suggesting that PC-12 cells culturing on dK had significantly higher axon–extension–related protein expression than those culturing on the dN scaffold.

### VEGF and HGF in dK could promote PC-12 axon extension

**Figure 7:**
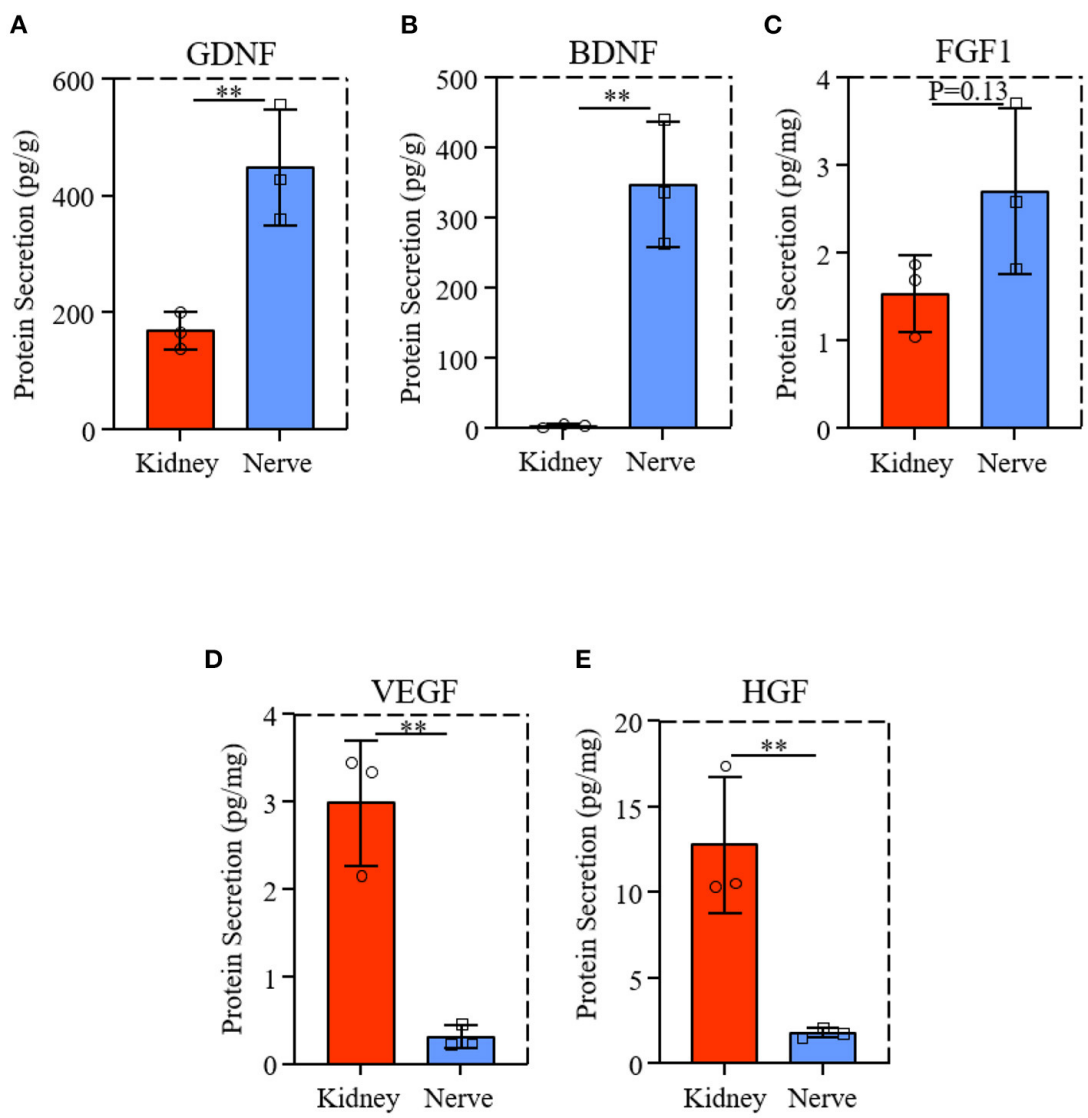
Neurotrophic factors and growth factors expression from dK and dN; **(A–C)** ELISA result of GDNF, BDNF, FGF1; **(D,E)** VEGF and HGF content in decellularized tissues. **P ≤ 0.01.

We evaluated the GDNF and BDNF protein levels from dK and dN, the neurotrophic factors for axon growth, suggesting that dN had a significantly higher expression of GDNF and BDNF than dK (Figures 7A,B). FGF1 is a growth factor that regulate migration, proliferation and neural functions of various cells; however, there was no significant difference for the FGF1 level between two decellularized tissues (Figure 7C, *P* = 0.13), indicating the existence of other potential biomolecular cues in dK that promotes the cellular axon extension and growth. Therefore, we further measured VEGF and HGF levels from both dK and dN, since VEGF (23) and HGF (24) are both angiogenic factors that have been reported to work on the nervous system directly or indirectly, as shown in Figures 7D,E. dK showed a much higher secretion of VEGF and HGF than did dN, which indicates that the axon extension for PC-12 cells may be partially or initially regulated by angiogenesis.

Taken together, the results above suggest that dK showed a similar effect on PC-12 cell proliferation, cell survival, and morphology compared to dN, but played a positive role in higher axon growth and the extension of neural-like cells, which may be partially mediated by high VEGF and HGF expression, indicating that the decellularized kidney could be a promising bio-scaffold to promote the regeneration of the peripheral nerve instead of the acellular nerve.

## Discussion

In this study, we evaluated two rat decellularized tissues—kidney and peripheral nerve—for nerve regeneration *in vitro* by observing PC-12 cell behaviors. Tissue-derived dECM scaffolds are made from specific tissues and have a natural 3D architecture of the full tissue by removing immunogenic cellular components while keeping a non-immunogenic ECM. These tissues go through a decellularization process. The acellular ECM scaffolds can be seeded with specific cells to produce tissue-engineered transplants (25–27). Tissue-derived dECM scaffolds serve as reservoirs for site-specific bioactive compounds and cell-matrix interactions, providing several benefits for tissue regeneration. In nerve-tissue engineering, decellularized nerve scaffolds have not only a native architecture for guiding cell migration and directing axonal trajectories, but also retain ECM components, such as glycosaminoglycans and proteoglycans. They are shown to affect neural stem cells proliferation and regulate synapse formation. Nonetheless, size availability, the efficiency of decellularization, and nutrients are still the main limitations on the use of acellular nerve.

Recently, more researchers have focussed on developing novel bio-scaffolds for heterotopic transplantation. It has been reported that SIS has been widely used in bone and skin tissue engineering (20, 28), which showed the potential application of other tissue-derived dECMs. Native kidney ECM has been shown to provide a scaffold for cell seeding and a niche for stem cells to differentiate into a full renal organ, which is important for kidney development and repair (29). The richness of ECM components in decellularized kidney, such as laminin and various types of collagens, also gives them functionality in re-epithelialization, granulation tissue formation, and neovascularization (30). The aim of this study is to investigate the potential possibility of acellular rat kidney for nerve regeneration *in vitro*. Although decellularized kidney shows similar efficiency for PC-12 cell proliferation and morphology compared to acellular nerve (Figures 3, 4), it has significant promotion for axon growth and extension-associated genes and protein expression (Figures 5, 6). These results may be caused by a difference of porosity and pore size between dK and dN as physical properties may mechanically affect cell growth and nutrient transport (31, 32).

Additionally, we quantified the expression of BDNF, GDNF, and FGF1 from both decellularized rat tissues using ELISA assay. Interestingly, these results were the complete opposite of our cellular-growth results—dN had a significantly higher expression level of neurotrophic factors and growth factors than dK. Similar data was reported previously (33), which theoretically contributes to the high protein expression of axon growth.

In order to clarify the potential mechanism of acellular rat kidney in regulating PC-12 cell behaviors, we further examined kinds of growth factors in dK and dN. More and more studies have shown that HGF and VEGF have abundant expression in various tissue-derived dECMs (34, 35), especially in decellularized kidney (36, 37), and have proved responsible for providing nutrition to newborn neurofilament extensions (24, 38, 39). To identify these growth factors involved in axon proliferation and extension, the dK and dN lysates were further evaluated by ELISA assay. This showed that dK had significantly higher VEGF and HGF expression compared to dN, which indicates that acellular kidney may promote axon growth partially through HGF and VEGF expression. Further, FGF and VEGF are the two most essential growth factors in the decellularized kidney matrix with the ability to induce the growth of new blood vessels. HGF is involved in tissue regeneration and other biological processes. The retention of these critical cytokines created a more realistic microenvironment for cell growth, whereas the artificial *in vitro* model did not. The cellular results in this study confirmed that acellular kidney showed high potential in axon growth, including high NF-H and GAP-43 expression, which also provides a novel strategy for nerve-tissue engineering by not only using acellular nerve, but also selecting other decellularized tissues, such as acellular kidney.

## Conclusion

In summary, we prepared and detected decellularized rat kidney and peripheral nerve. Material characterization was analyzed using SEM, pore size, and porosity. The effect of dK and dN on PC-12 cell proliferation, cell survival, and cell arrangement *in vitro* was further analyzed. Additionally, we examined axon-associated genes and protein expression, both of them showed significantly higher for PC-12 cells cultring on dK compared to the dN group. The lower expression of neurotrophic factors but higher growth-factor expression indicates that axon growth and extension for PC-12 cells may be partially mediated by VEGF and HGF expression from decellularized kidney, providing more bio-scaffold selection in nerve-tissue repair.

## Data availability statement

The raw data supporting the conclusions of this article will be made available by the authors, without undue reservation.

## Ethics statement

The animal study was reviewed and approved by Institutional Animal Care and Use Committee of Ningbo University (No. NBU20210032).

## Author contributions

KY, AH, and YuH: conceptualization. KY, XQ, MW, and ZC: data curation. YuH and KX: formal analysis. KY, AH, YiH, QS, YuH, and KX: methodology. YuH, PW, and JY: resources. PW and JY: supervision. PW, YuH, and KX: validation. KY and YuH: writing—original draft. KX, PW, and JY: writing—review & editing. PW and YuH: funding acquisition. All authors have read and agreed to the published version of the manuscript.

## Funding

This study was partially supported by the Zhejiang Medical Science and Technology Project (Grant Nos. 2022KY1101, 2019KY569, and 2022KY311), the National Key Research and Development Program of China (Grant No. 2018YFA0703000), and the National Natural Science Foundation of China (Grant No. 52075482).

## Conflict of interest

The authors declare that the research was conducted in the absence of any commercial or financial relationships that could be construed as a potential conflict of interest.

## Publisher's note

All claims expressed in this article are solely those of the authors and do not necessarily represent those of their affiliated organizations, or those of the publisher, the editors and the reviewers. Any product that may be evaluated in this article, or claim that may be made by its manufacturer, is not guaranteed or endorsed by the publisher.
